# White matter hyperintensities are a prominent feature of autosomal dominant Alzheimer’s disease that emerge prior to dementia

**DOI:** 10.1186/s13195-022-01030-7

**Published:** 2022-06-29

**Authors:** Dorothee Schoemaker, Maria Clara Zanon Zotin, Kewei Chen, Kay C. Igwe, Clara Vila-Castelar, Jairo Martinez, Ana Baena, Joshua T. Fox-Fuller, Francisco Lopera, Eric M. Reiman, Adam M. Brickman, Yakeel T. Quiroz

**Affiliations:** 1 Department of Psychiatry, Massachusetts General Hospital, Harvard Medical School, Boston, MA USA; 2 J. Philip Kistler Stroke Research Center, Department of Neurology, Massachusetts General Hospital, Harvard Medical School, Boston, MA USA; 3 Center for Imaging Sciences and Medical Physics, Department of Medical Imaging, Hematology and Clinical Oncology, Ribeirão Preto Medical School, University of São Paulo, Ribeirão Preto, SP Brazil; 4 Banner Alzheimer’s Institute, Phoenix, AZ USA; 5 Taub Institute for Research On Alzheimer’s Disease and the Aging Brain, Vagelos College of Physicians and Surgeons, Columbia University, New York, NY USA; 6 Department of Psychological and Brain Sciences, Boston University, Boston, MA USA; 7 Grupo Neurociencias de Antioquia, Universidad de Antioquia, Medellín, Colombia; 8 Gertrude H. Sergievsky Center and Department of Neurology, Vagelos College of Physicians and Surgeons, Columbia University, New York, NY USA; 9 Department of Neurology, Massachusetts General Hospital, Harvard Medical School, MB Boston, USA

**Keywords:** Autosomal-dominant Alzheimer’s disease, PSEN1, White matter hyperintensities, Cognition, Dementia, Cerebral microbleeds

## Abstract

**Background:**

To promote the development of effective therapies, there is an important need to characterize the full spectrum of neuropathological changes associated with Alzheimer’s disease. In line with this need, this study examined white matter abnormalities in individuals with early-onset autosomal dominant Alzheimer’s disease, in relation to age and symptom severity.

**Methods:**

This is a cross-sectional analysis of data collected in members of a large kindred with a *PSEN1* E280A mutation. Participants were recruited between September 2011 and July 2012 from the Colombian Alzheimer’s Prevention Initiative registry. The studied cohort comprised 50 participants aged between 20 and 55 years, including 20 cognitively unimpaired mutation carriers, 9 cognitively impaired mutation carriers, and 21 non-carriers. Participants completed an MRI, a lumbar puncture for cerebrospinal fluid collection, a florbetapir PET scan, and neurological and neuropsychological examinations. The volume of white matter hyperintensities (WMH) was compared between cognitively unimpaired carriers, cognitively impaired carriers, and non-carriers. Relationships between WMH, age, and cognitive performance were further examined in mutation carriers.

**Results:**

The mean (SD) age of participants was 35.8 (9.6) years and 64% were women. Cardiovascular risk factors were uncommon and did not differ across groups. Cognitively impaired carriers [median, 6.37; interquartile range (IQR), 9.15] had an increased volume of WMH compared to both cognitively unimpaired carriers [median, 0.85; IQR, 0.79] and non-carriers [median, 1.07; IQR, 0.71]. In mutation carriers, the volume of WMH strongly correlated with cognition and age, with evidence for an accelerated rate of changes after the age of 43 years, 1 year earlier than the estimated median age of symptom onset. In multivariable regression models including cortical amyloid retention, superior parietal lobe cortical thickness, and cerebrospinal fluid phospho-tau, the volume of WMH was the only biomarker independently and significantly contributing to the total explained variance in cognitive performance.

**Conclusions:**

The volume of WMH is increased among individuals with symptomatic autosomal-dominant Alzheimer’s disease, begins to increase prior to clinical symptom onset, and is an independent determinant of cognitive performance in this group. These findings suggest that WMH are a key component of autosomal-dominant Alzheimer’s disease that is closely related to the progression of clinical symptoms.

## Background

Cerebrovascular changes, including white matter hyperintensities (WMH), cerebral microbleeds, and lacunar infarcts, are prominent features of Alzheimer’s disease (AD) [1], contributing to the rate of cognitive decline across disease stages [2–4]. In patients with AD, WMH typically precede the onset of clinical symptoms [5, 6] and become more prominent over time, with advancing disease severity [5, 7]. White matter hyperintensities are also observed in individuals with early-onset autosomal-dominant AD (ADAD) [5, 8–10], a population characterized by a younger age of symptom onset and a low frequency of vascular risk factors. This evidence suggests that the presence of WMH is not solely explained by aging or age-related comorbidities but is rather implicated in AD pathogenesis [11].

Yet, little is known about the trajectory of WMH from the asymptomatic to symptomatic disease stages. Past efforts to characterize relationships among age, cognitive symptoms, and WMH in patients with late-onset sporadic AD are confounded by the high frequency of age-related comorbidities in this population [12]. Investigating these associations in younger individuals with a deterministic genetic mutation for AD, presenting low levels of cardiovascular risk factors, offers a unique opportunity to determine whether the presence of WMH is a core independent feature of AD and characterize the temporal relationship between the emergence of WMH and clinical symptom onset.

Relying on data from a homogeneous cohort of individuals carrying the Presenilin 1 (*PSEN1*) E280A mutation leading to ADAD, this study examined the severity of WMH in relation to age and cognitive symptoms. Carriers of the *PSEN1* E280A have a well-defined and relatively homogenous disease course, with a median age of cognitive impairment at 44 years (95% C.I. = 43, 45 years) [13]. We hypothesized that carriers of the *PSEN1* E280A mutation would have greater WMH volume than non-carriers, which would relate to both older age and worsening cognitive performance.

## Methods

### Study design and participants

Participants were enrolled in this study between September 2011 and July 2012 from the Alzheimer’s Prevention Initiative (API) registry, a registry including members of a large Colombian kindred with the *PSEN1* E280A mutation leading to early-onset ADAD.

Participants included in this study ranged in age from 20 to 55 years. The investigation was performed in a double-blind fashion and genotyping for the *PSEN1* E280A mutation was performed after the completion of data collection to determine group status (i.e., carrier versus non-carrier). The study sample included a total of 50 individuals, comprising 21 non-carriers, 20 cognitively unimpaired mutation carriers, and 9 cognitively impaired mutation carriers. Participants completed a magnetic resonance imaging (MRI) scan, a lumbar puncture for cerebrospinal fluid (CSF) collection, a [^18^F] florbetapir positron emission tomography (PET) for in vivo quantification of amyloid burden, and a neurological and neuropsychological evaluation. All participants provided informed consent. Study procedures were carried in accordance with ethical standards from the Helsinki Declaration and were approved by the Bioethics Committee of the University of Antioquia (Medellín, Colombia) and the Institutional Review Board of the Massachusetts General Hospital (USA).

### Clinical and cognitive assessment

Neurological and neuropsychological evaluations were performed at the University of Antioquia. Functional status was determined based on clinical evaluations and rated on the Functional Assessment Staging Tool (FAST) [14]. Global cognitive functioning was assessed with the Mini-Mental State Examination (MMSE) [15]. Memory function was assessed with the Consortium to Establish a Registry for Alzheimer’s Disease (CERAD) Word List Learning subtest [16]. A CERAD Word List Learning Composite score was computed by adding performance on the total immediate recall, delayed recall, and recognition. Cardiovascular risk factors, including high blood pressure, body mass index (BMI), smoking status, and diabetes mellitus status, were assessed in all participants using standard clinical examination procedures.

### Magnetic resonance imaging acquisition

Participants completed an MRI scan at the Hospital Pablo Tobón Uribe (Medellín, Colombia), on a 1.5-T Avanto Siemens scanner. The MRI protocol included a T1 MPRAGE [repetition time (TR) = 2.4 s, echo time (TE) = 0.00361 s, flip angle (FA) = 8, resolution = 1 mm × 1.2 mm × 1.2 mm], a T2 FLAIR [TR = 9.0 s, TE = 0.095 s, FA = 150, resolution = 0.45 mm × 0.45 mm × 7.5 mm], a T2* [TR = 0.65 s, TE = 0.026 s, FA = 20, resolution = 0.45 mm × 0.45 mm × 7.5 mm], and a T2-TSE [TR = 3.0 s, TE = 0.097 s, FA = 150, resolution = 0.94 mm × 0.94 mm × 3.0 mm].

### Quantification of white matter lesions and cortical thickness

Anatomical scans were processed with the FreeSurfer volumetric pipeline (version 6.0) to obtain the estimated total intracranial volume (eTIV) [17]. Areas of WMH were automatically segmented on FLAIR images using a lesion prediction algorithm with a 0.5 lesion probability threshold as implemented in the LST toolbox (v. 3.0.0) for SPM [18]. The WMH volume was normalized to the eTIV to account for interindividual variations in brain size. Cerebral microbleeds and lacunes of presumed vascular origin were identified on MRI images and counted across the brain by a neuroradiologist (M.C.Z.Z) following published consensus guidelines (STRIVE) [19].

For quantification of lobe-specific WMH volume, the Wake Forest lobar atlas [20] was linearly aligned to native T2 FLAIR images, allowing a parcellation of these images into six lobar masks (periventricular/deep, frontal, limbic, temporal, parietal, occipital). The volume of segmented WMH in each lobe was extracted using the lobar masks and then normalized to the respective total lobar volume to account for variations in lobar volumes.

To obtain a measure of neurodegeneration, cortical thickness in the superior parietal lobe was extracted using a FreeSurfer-based pipeline (version 6.0), as previously described [21–23]. The cortical thickness metric was averaged between the left and right hemispheres. Previous research demonstrates that cortical thickness in this brain region is particularly sensitive to early changes in individuals with ADAD due to the *PSEN1* E280A mutation [21, 22].

### In vivo quantification of amyloid burden on positron emission tomography

To estimate global cortical amyloid burden, participants completed a [^18^F] florbetapir PET on a Siemens Biograph 16 HiRez PET/CT scanner at the Banner Alzheimer’s Institute (Arizona, USA). Three individuals, including one mutation carrier and two non-carriers, did not complete the amyloid PET scan. Briefly, images were acquired following the intravenous injection of 10 mCi of [^18^F] florbetapir radiotracers, a 50-min radiotracer uptake period, a 10-min emission scan, and a CT scan for correction of radiation attenuation. Images were reconstructed using an iterative algorithm, with attenuation–correction and a 5-mm full-width-at-half-maximum Gaussian filter.

Using a SPM12‐based pipeline, PET images were first aligned to a standard brain atlas. The quantification of the global cortical amyloid burden was obtained following a previously described method [24]. In brief, cortical-to-pontine standard uptake value ratios (SUVRs) were extracted and averaged across six pre-defined cortical regions of interest regrouping frontal, temporal, parietal, anterior cingulate, posterior cingulate, and precuneus regions.

### Cerebrospinal fluid analysis

Lumbar punctures to collect CSF samples were performed at the University of Antioquia in the morning after an overnight fast. Samples were processed, stored in polypropylene tubes, frozen at − 80 °C, and shipped to the Knight Alzheimer’s Disease Research Center Biomarker Core at Washington University in St. Louis (Missouri, USA) for analyses. CSF concentrations of amyloid-beta 42 (CSF Aβ_42_) and phospho-tau (CSF ptau) were quantified using a Luminex xMAP (Bio Rad, California, USA) bead-based method, following a previously described procedure [22, 25].

### Statistical analyses

Statistical analyses were performed using SPSS version 27.0 (SPSS Inc, Chicago, IL) and R version 3.6.3 (R Foundation for Statistical Computing, Vienna, Austria). Cognitively impaired and unimpaired *PSEN1* E280A mutation carriers were compared to non-carriers on demographic and clinical variables using one-way ANOVAs for continuous variables and chi-square tests for dichotomous variables. Group differences on the FAST were assessed using the Kruskal–Wallis test. Performance on the MMSE and the CERAD Word List Learning Composite score was contrasted across groups using ANCOVAs adjusting for age and education. Group differences on neuroimaging and fluid biomarkers were assessed using generalized linear models with a linear distribution for continuous variables and a Poisson distribution for count variables (i.e., count of cerebral microbleeds and lacunes), while adjusting for age. Post hoc pairwise comparisons across groups were assessed using the Bonferroni correction for multiple comparisons.

Associations between age and normalized WMH (nWMH) volume were assessed using a regression model with a quadratic fit. A piecewise regression was further applied to identify the breakpoint marking the age at which the slope significantly steepens. To allow a visual comparison of biomarkers (nWMH volume, PET measure of cortical amyloid, superior parietal lobe cortical thickness, CSF Aβ_42_, and CSF ptau) in their relationship to age in mutation carriers, each individual biomarker was transformed into a *Z*-score using the mean of the non-carrier group. Transformed *Z*-scores, reflecting the extent of biomarker abnormality in carrier relative to non-carrier individuals, were then plotted against age.

The association between cognition and nWMH volume in mutation carriers was first assessed in partial correlations, adjusting for education level. The independent contribution of nWMH volume with regard to the performance of mutation carriers on the MMSE and CERAD Word List Learning Composite was examined with multivariable linear regression models that included other relevant biomarkers (superior parietal lobe cortical thickness, global cortical amyloid, and CSF ptau) and education as additional covariables. To avoid issues associated with collinearity, CSF Aβ_42_ was not included in these models. However, exploratory analyses confirmed that findings were equivalent whether using CSF or PET measures of beta amyloid. Finally, the relative contribution of each predictor to the overall explained variance in cognitive scores, while accounting for the intercorrelation between regressors, was examined using the lmg metric obtained with the “relaimpo” package in R [26].

## Results

### Group differences in demographic, clinical, and biomarker characteristics

A summary of demographic, clinical, and biomarker characteristics across groups is presented in Table 1. Cognitively impaired mutation carriers had greater nWMH volume than both cognitively unimpaired carriers and non-carriers. In contrast, nWMH volume did not differ between cognitively unimpaired carriers and non-carriers. The count of cerebral microbleeds or lacunes, which were uncommon across the three groups, did not differ across groups. One single lobar cerebral microbleed was found in the sample, in a cognitively unimpaired mutation carrier individual, and two deep lacunes were found in a non-carrier individual.

**Table 1:** Group differences in demographic, clinical, and biomarker characteristics

	**Non-carriers**^**a**^ **(*N* = 21)**	**Cognitively unimpaired carriers**^**b**^ **(*n* = 20)**	**Cognitively impaired carriers**^**c**^ **(*n* = 9)**	**Sig**
**Demographics**				
*Age — mean (SD)*	33.8 (8.7)	32.6 (8.8)	47.3 (3.5)	c > a***, c > b***
*Education — mean (SD)*	11.4 (3.3)	12.1 (2.6)	8.7 (4.1)	b > c*
*Female — n (%)*	14 (66.7)	12 (60.0)	6 (66.7)	*ns*
**Cardiovascular risk factors**				
*High blood pressure — n (%)*	0 (0)	0 (0)	0 (0)	*ns*
*Diabetes — n (%)*	0 (0)	0 (0)	0 (0)	*ns*
*Smoking — n (%)*	1 (4.8)	2 (10.0)	2 (22.2)	*ns*
*BMI — mean (SD)*	23.6 (2.8)	23.3 (3.8)	23.8 (2.9)	*ns*
**Cognitive status**				
*FAST — median (IQR)*	1 (0.0)	1 (0.0)	4 (2)	c > a***, c > b***
*MMSE — mean (SD)*	29.8 (0.4)	29.8 (0.7)	23.3 (3.2)	c < a***, c < b***
*CERAD Word List Learning Composite — mean (SD)*	36.6 (4.4)	37.3 (5.4)	10.8 (5.5)	c < a***, c < b***
**Neuroimaging markers**				
nWMH Vol. *1000 — median (IQR)	1.07 (0.71)	0.85 (0.79)	6.37 (9.15)	c > a***, c > b***
*Cerebral microbleeds — median (IQR)*	0.0 (0)	0.0 (0)	0.0 (0)	*ns*
*Lacunes — median (IQR)*	0.0 (0)	0.0 (0)	0.0 (0)	*ns*
*Superior parietal lobe cortical thickness — median (IQR)*	2.19 (0.17)	2.19 (0.17)	1.80 (0.23)	c < a***, c < b***
*Cortical amyloid PET (SUVR) — median (IQR)*	0.80 (0.09)	0.95 (0.27)	1.13 (0.09)	a < b***, a < c***
**Cerebrospinal fluid markers**				
*CSF Aβ*_*42*_* — median (IQR)*	495.1 (136.1)	357.8 (190.9)	233.6 (111.2)	a > b*, a > c**
*CSF ptau — median (IQR)*	18.82 (7.73)	36.25 (43.63)	69.40 (66.04)	a < b***, a < c***

*BMI* Body Mass Index, *SD* Standard deviation, *IQR* Interquartile range, *FAST* Functional Assessment Staging Tool, *MMSE* Mini-Mental State Exam, *nWMH Vol.* Normalized white matter hyperintensity volume, *SUVR* Standardized uptake value ratio, *PET* Positron emission tomography, *CSF* Cerebrospinal fluid, *ptau* Phospho-tau, *Aβ* Amyloid-beta. *p < 0.05, **p < 0.01, ***p < 0.001

Non-carriers had lower cortical amyloid burden on PET, greater levels of CSF Aβ_42_, and lower levels of CSF ptau than both cognitively unimpaired and impaired mutation carriers, whereas these measures did not differ between cognitively unimpaired and impaired carriers. Cognitively impaired mutation carriers had reduced cortical thickness in the superior parietal lobe compared to both non-carriers and cognitively unimpaired carriers, whereas no differences were found between cognitively unimpaired mutation carriers and non-carriers.

Cognitively impaired mutation carriers had lower scores on the FAST, MMSE, and CERAD Word List Composite than both cognitively unimpaired carriers and non-carriers. Cognitively unimpaired mutation carriers did not significantly differ from non-carriers on these cognitive measures. Cardiovascular risk factors were rare in this cohort and did not differ across groups.

### Associations of age with white matter hyperintensity volume

**Fig. 1:**
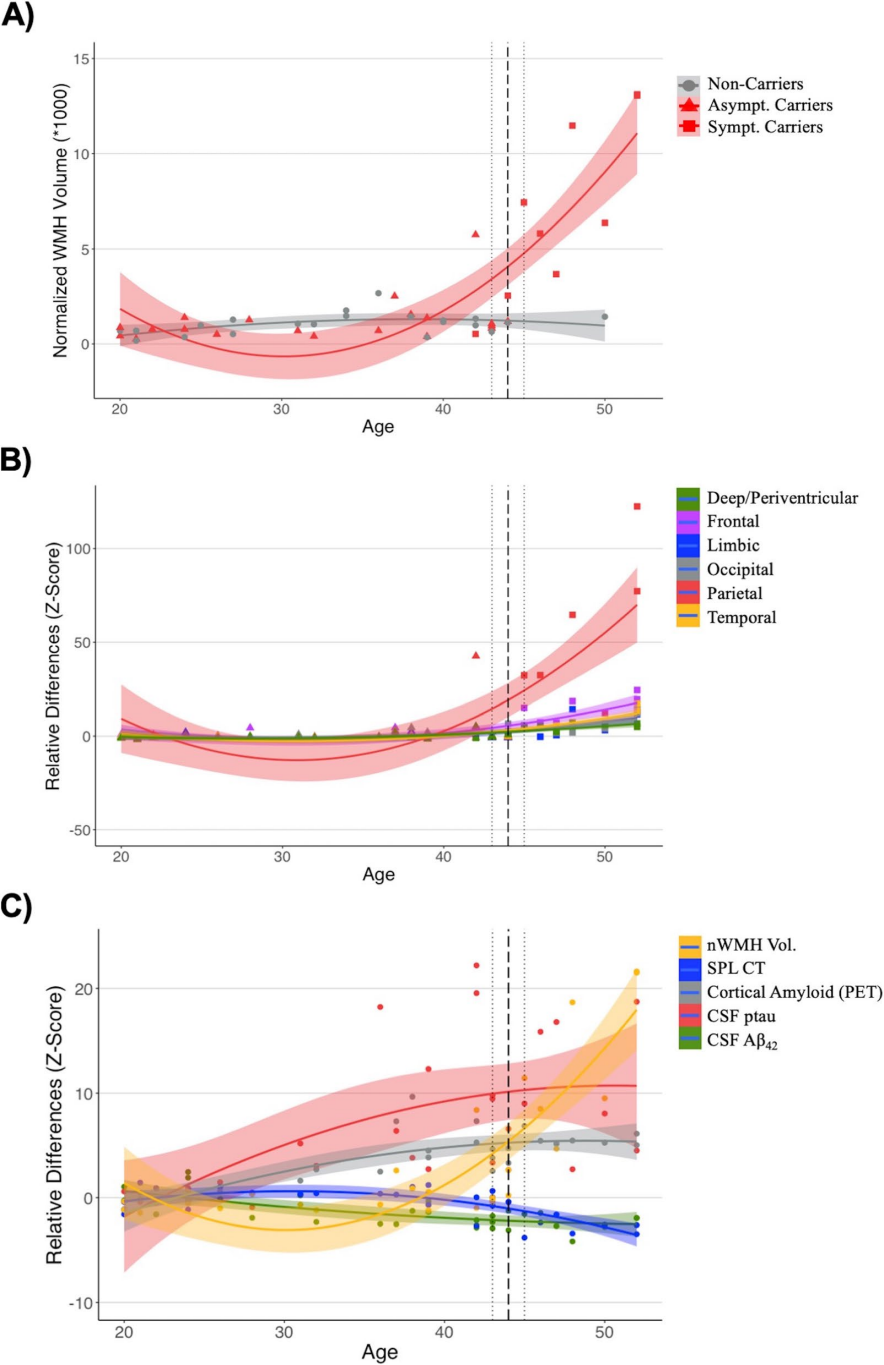
Associations of age with **A** global normalized white matter hyperintensity volume (WMH) in *PSEN1* E280A mutation carriers and non-carriers; **B** relative differences in lobe-specific normalized WMH volume in *PSEN1* E280A mutation carriers; **C** relative differences across quantified biomarkers in *PSEN1* E280A mutation carriers. All regression curves were fitted with a quadratic model and the 95% confidence intervals. In plots **B** and **C**, *Z*-scores reflecting relative differences were obtained using the mean of non-carrier individuals. Cortical amyloid (PET), measure of global cortical amyloid accumulation from positron emission tomography (PET) expressed in standardized uptake value ratio (SUVR); CSF, cerebrospinal fluid; ptau, phospho-tau; *Aβ*, amyloid-beta; SPL CT, superior parietal lobe cortical thickness; nWMH, normalized white matter hyperintensity volume. The dashed vertical line represents the median age of symptom onset in this clinical sample (i.e., 44 years) and the two vertical dotted lines represent 95% confidence intervals (i.e., 95% C.I. = 43, 45 years)

Age was strongly associated with the nWMH volume (Fig. 1A) in mutation carriers (*R*^2^ = 0.74, *p* < 0.001), but not in non-carriers (*R*^2^ = 0.26, *p* > 0.05). In mutation carriers, the point of inflection of the slope was found at 43 years, 1 year earlier than the estimated median age of clinical symptom onset in this population. The relative difference in lobe-specific nWMH volume between carrier and non-carrier individuals was particularly pronounced in the parietal lobe with advancing age (Fig. 1B). When examining relationships between age and differences in biomarkers in carriers relative to non-carriers (Fig. 1C), an increase in cortical amyloid and CSF ptau, as well as a decrease in CSF Aβ_42_, is noted at an early stage in mutation carriers. Yet, the magnitude of these differences appeared to plateau prior to the estimated median age of clinical symptom onset and to remain relatively stable past that point. Changes in nWMH volume followed a different pattern and became prominent only at a later stage, with a rapid acceleration in close proximity to the estimated median age of clinical symptom onset and a continued increase with advancing age.

### Associations of cognition with white matter hyperintensity volume

**Fig. 2:**
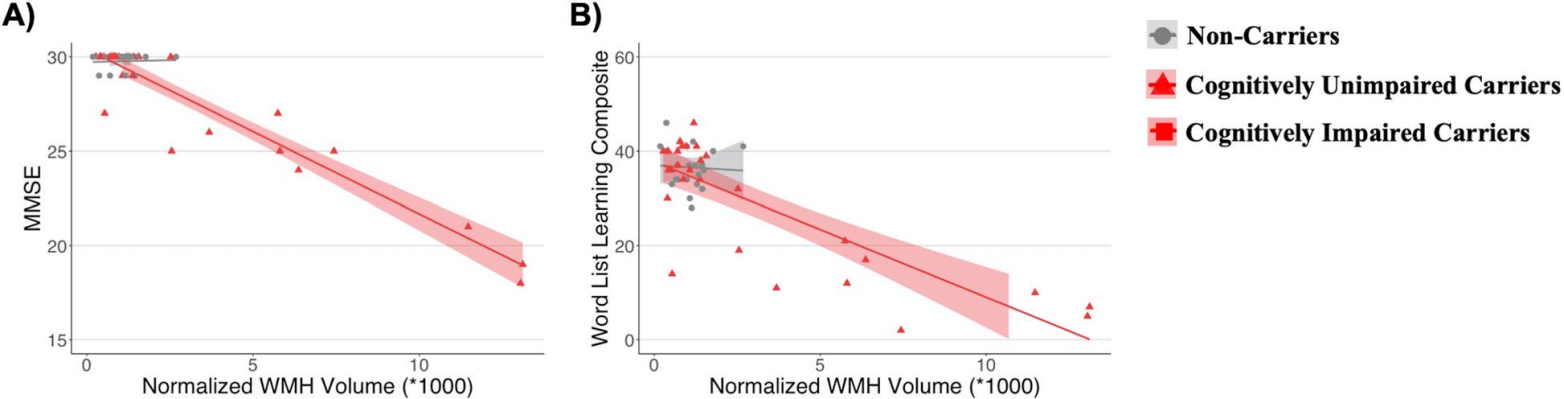
Associations between normalized white matter hyperintensity (WMH) volume, **A** the Mini-Mental State Exam (MMSE) score, and **B** the CERAD Word List Learning Composite score in *PSEN1* E280A mutation carriers (red) and non-carriers (gray)

In partial correlations adjusting for education, greater nWMH volume was strongly associated with worse performance on the MMSE (*r* =  − 0.95, *p* < 0.001; Fig. 2A) and the CERAD Word List Composite (*r* =  − 0.85, *p* < 0.001; Fig. 2B) in mutation carriers. These associations were not significant in non-carrier individuals (MMSE: *r* = 0.05, *p* > 0.05; CERAD Word List Learning Composite: *r* =  − 0.06, *p* > 0.05).

**Fig. 3:**
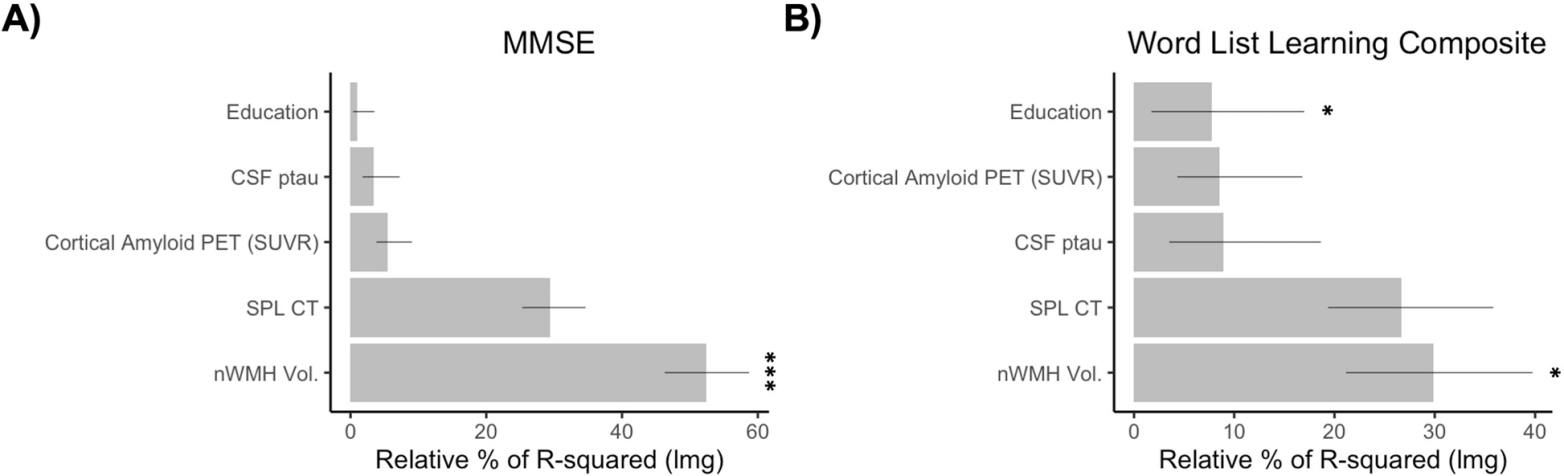
Bar graphs representing the relative contribution of each biomarker to the overall explained variance in performance of *PSEN1* E280A mutation carriers on **A** the Mini-Mental State Exam (MMSE) score and **B** the CERAD Word List Learning Composite score. The relative contribution of each regressor is quantified using the LMG metric computed with the R package “relaimpo” (U. Grömping, 2006). Lines represent 95% confidence intervals after 1000 bootstrapping replications. CSF, cerebrospinal fluid; ptau, phospho-tau; SPL CT, superior parietal lobe cortical thickness; nWMH Vol., normalized white matter hyperintensity volume. *p < 0.05, **p < 0.01, ***p < 0.001

The results of linear regression models assessing the independent association of biomarkers with the MMSE score and the CERAD Word List Composite score are presented in Table 2. Overall, across the two models, the nWMH volume was the only biomarker independently associated with measures of cognitive function. When evaluating the relative and independent contribution of each biomarker to the total explained variance in cognitive scores, results confirmed that the nWMH volume independently and significantly accounted for the largest proportion of total explained variance in MMSE score and memory performance (Fig. 3).

**Table 2:** Associations of biomarkers with cognitive measures in *PSEN1* mutation carriers

	**MMSE** **total score**				**CERAD Word List Learning Composite**			
**Model summary**	**Adjusted *R*-squared: 0.90 (*p* < 0.001)**				**Adjusted *R*-squared: 0.78** **(*p* < 0.001)**			
	**Std** **beta**	**95% CI**		***p*-value**	**Std** **beta**	**95% CI**		***p*-value**
**nWMH volume**	− 1.01	− 1.28	− 0.74	< .001***	− 0.54	− 0.94	− 0.14	0.011*
**Cortical amyloid (PET)**	0.06	− 0.11	0.23	0.484	− 0.05	− 0.31	0.20	0.658
**CSF ptau**	− 0.09	− 0.25	0.07	0.273	− 0.18	− 0.42	0.07	0.148
**Cortical thickness SPL**	− 0.08	− 0.35	0.19	0.566	0.21	− 0.19	0.61	0.298
**Education**	0.09	− 0.05	0.23	0.214	0.24	0.03	0.44	0.027*

*Std. beta* Standardized beta coefficient, *CI* Confidence intervals, *MMSE* Mini-Mental State Exam, *nWMH volume* Normalized white matter hyperintensity volume, *PET* Positron emission tomography, *CSF* Cerebrospinal fluid, *ptau* Phospho-tau, *SPL* Superior parietal lobe. *p < 0.05, **p < 0.01, ***p < 0.001

## Discussion

This study examined the presence of WMH in relation to age and cognition in a homogeneous cohort of cognitively unimpaired and impaired carriers of the *PSEN1* E280A mutation. Our results highlighted an increased burden of WMH in cognitively impaired mutation carriers in the absence of significant cardiovascular risk factors. The increase in WMH was strongly associated with age and cognition. Consistent with previous findings [5, 9], these results emphasize the relevance of WMH as a core neuroimaging feature of autosomal-dominant AD.

The volume of WMH was increased in cognitively impaired mutation carriers compared with both cognitively unimpaired mutation carriers and non-carriers. In contrast, cortical amyloid burden on PET and CSF ptau was increased, and CSF Aβ_42_ decreased, in both cognitively unimpaired and cognitively impaired mutation carriers relative to non-carriers but did not differ between cognitively unimpaired and impaired mutation carriers. These findings suggest that, while changes in biomarkers of amyloid and tau pathology occur at an early and presymptomatic stage in mutation carriers, these changes appear to stabilize as the disease further progresses from the asymptomatic to symptomatic stages. In contrast, changes in WMH volume arise at a later age in mutation carriers, becoming prominent during the early symptomatic disease stages. As opposed to previous reports [9], cerebral microbleeds were rare in this cohort and found in only one cognitively unimpaired mutation carrier. The low prevalence of cerebral microbleeds in this cohort may be explained by the limited resolution of images associated with the MRI field strength and scanning protocol. The low prevalence of cerebral microbleeds could also reflect characteristics specific to the studied cohort of mutation carriers, including the genotype [27] and overall mild disease severity.

When analyzing relationships between biomarkers and age, the volume of WMH began to sharply increase around the age of 43 years in mutation carriers, an age close to but preceding the estimated median age of cognitive symptom onset in this clinical population (i.e., 44 years, 95% CI = 43, 45 years) [13], and continued to increase exponentially with advancing age. Age-related changes in superior parietal lobe cortical thickness followed an inverse pattern, with a decrease in volume around the median age of clinical symptom onset and further decline with advancing age, although the effect was of lesser magnitude than that observed with the WMH volume. In contrast, changes in cortical amyloid burden on PET, CSF ptau, and CSF Aβ_42_ emerged at an earlier age in mutation carriers but appeared to stabilize around the estimated median age of clinical symptom onset, with only limited progression during the symptomatic disease stages. The relationships between age and changes in biomarkers of amyloid or tau pathology reported in this study are consistent with previous reports in carriers of the *PSEN1* E280A mutation [24, 25]. When contrasting lobe-specific changes in WMH volume, the parietal lobe appeared to be the most severely affected region [24]. This observation is noteworthy because several studies have reported a specific involvement of posterior WMH distribution in both hereditary and sporadic forms of AD [5, 28]. While it remains unclear why several AD-associated biological changes converge in posterior brain regions, this observation raises the possibility of anatomical and mechanistic interactions among AD pathological features [29–32]. While WMH has typically been linked to cerebrovascular dysfunction in healthy aging individuals, some authors suggested that WMH in individuals with AD may instead reflect “Wallerian degeneration” resulting from AD neuropathology [33], notably cortical tau burden [34]. In patients with sporadic AD, baseline levels of CSF Aβ_1–42_ predicted an increase in WMH volume, suggesting a potential role of beta amyloid in driving white matter pathology [35]. Loss of white matter integrity has also been associated with tau burden in patients with sporadic AD [36], although inconsistently [31]. Some evidence instead suggests that the appearance of WMH precedes tau-mediated neurodegeneration in the AD disease trajectory [28, 35, 37], and changes in white matter and blood–brain barrier integrity have been reported at preclinical disease stages in patients with both sporadic and autosomal-dominant AD [38, 39]. The distribution of WMH also appears to follow perfusion patterns, beginning mostly in lower perfusion areas [40]. As such, it has been argued that cerebrovascular dysfunctions might act as a primary pathological process in AD, potentially promoting tau hyperphosphorylation rather than resulting from it [41]. The etiology of WMH in AD is still a matter of debate and additional work is needed to characterize pathophysiological mechanisms underlying the emergence of WMH in autosomal-dominant and sporadic AD [42]. Such efforts may provide important insights into the complex pathogenesis of AD, with a potential to highlight novel avenues for treatment and prevention.

When characterizing relationships between WMH volume and cognition in mutation carriers, strong associations were found with two separate measures of cognitive function: the MMSE and the CERAD Word List Learning. In multivariable linear regression models with other well-characterized disease biomarkers, including cortical thickness in the superior parietal lobe, CSF ptau, and cortical amyloid burden on PET imaging, WMH volume was the only biomarker independently and significantly predicting cognitive performance in mutation carriers. These results align with past research demonstrating an independent association between WMH and cognitive trajectory in aging individuals with normal cognition, mild cognitive impairment, and sporadic AD [43]. The current findings also underscore the limitations of certain frameworks, such as the amyloid-tau-neurodegeneration (ATN) [44], in accounting for the full severity of cognitive impairment, which ultimately mediates the individual, familial, community, and global public health impact of the disease.

Overall, the present results suggest that WMH is a clinically significant neuroimaging feature that is closely correlated with age and cognitive impairment in individuals with early-onset autosomal-dominant AD. These findings enhance our understanding of white matter abnormalities in AD, in relation to progressing disease severity. The clinical sample examined in this study was selected due to its high homogeneity and well-characterized disease course. However, as a consequence, our sample size was also relatively small. Future research should replicate these findings in larger and more heterogeneous samples to confirm the generalizability of these results to patients with ADAD due to other mutations. Our findings provide important information on relationships between WMH, age, and cognition in early-onset ADAD, a clinical sample not confounded by age-related comorbidities. However, due to the cross-sectional nature of this study, it remains challenging to determine if interindividual variability in biomarkers contributed to the obtained results or if the present findings truly represent a meaningful biological effect of ADAD. Studies using longitudinal designs in this clinical population are thus needed to characterize the trajectory of WMH volume changes in relation to age, cognitive decline, and other well-defined disease risk factors (e.g., APOE ε4 genotype). As the examination of the relationship between WMH and other key biomarkers of AD pathologies was outside the scope of the present study, future work is also still needed to evaluate the temporal and spatial overlap between WMH volume, beta amyloid, and tau pathology and characterize their interplay in contributing to cognitive deterioration.

## Conclusions

To conclude, the present study highlights important changes in WMH closely coinciding with the onset of clinical symptoms in carriers of the *PSEN1* E280A mutation. The increase in WMH volume in mutation carriers was not related to differences in cardiovascular risk factors or sociodemographic background. When assessed in combination with other well-established disease biomarkers, the burden of WMH was found to be a significant and independent predictor of cognitive impairment in mutation carriers. These findings emphasize the clinical significance of white matter changes in autosomal-dominant AD.

## Data Availability

The data that support the findings of this study are available on request from the corresponding author Y.T.Q. The data are not publicly available because they contain information that could compromise research participant privacy and anonymity.

## Abbreviations

WMH: White matter hyperintensities
AD: Alzheimer’s disease
ADAD: Autosomal-dominant AD
*PSEN1*: Presenilin 1
MRI: Magnetic resonance imaging
CSF: Cerebrospinal fluid
PET: Positron emission tomography
FAST: Functional Assessment Staging Tool
CERAD: Consortium to Establish a Registry for Alzheimer’s Disease
MMSE: Mini-Mental State Examination
BMI: Body mass index
eTIV: Estimated total intracranial volume
SUVRs: Standard uptake value ratios
Aβ_42_: Amyloid-beta 42
ptau: Phospho-tau
nWMH volume: Normalized WMH volume
SPL CT: Superior parietal lobe cortical thickness
